# Ferulic Acid Relieves the Oxidative Stress Induced by Oxidized Fish Oil in Oriental River Prawn (*Macrobrachium nipponense*) with an Emphasis on Lipid Metabolism and Gut Microbiota

**DOI:** 10.3390/antiox13121463

**Published:** 2024-11-28

**Authors:** Xin Liu, Cunxin Sun, Qunlan Zhou, Xiaochuan Zheng, Sufei Jiang, Aimin Wang, Yongquan Han, Gangchun Xu, Bo Liu

**Affiliations:** 1 Wuxi Fisheries College, Nanjing Agricultural University, Wuxi 214081, China; 2023213007@stu.njau.edu.cn (X.L.); suncx@ffrc.cn (C.S.); zhouql@ffrc.cn (Q.Z.); zhengxiaochuan@ffrc.cn (X.Z.); jiangsf@ffrc.cn (S.J.); 2 Key Laboratory of Aquatic Animal Nutrition and Health, Freshwater Fisheries Research Center, Chinese Academy of Fishery Science, Wuxi 214081, China; 3 Key Laboratory of Freshwater Fisheries and Germplasm Resources Utilization, Ministry of Agriculture and Rural Affairs, Freshwater Fisheries Research Center, Chinese Academy of Fishery Sciences, Wuxi 214081, China; 4 Yancheng Academy of Fishery Science, Yancheng 224051, China; zam--3@163.com; 5 Guangzhou Cohoo Biotechnology Co., Ltd., Guangzhou 510663, China; yhan@co-hoo.com

**Keywords:** ferulic acid, *Macrobrachium nipponense*, oxidized fish oil, lipid metabolism, gut microbiota

## Abstract

To investigate the potential of ferulic acid (FA) in attenuating the deleterious effects of oxidized fish oil (OF) on *Macrobrachium nipponense*, four experimental diets were formulated: 3% fresh fish oil (CT group, peroxide value: 2.2 mmol/kg), 3% oxidized fish oil (OF group, peroxide value: 318 mmol/kg), and 3% OF with an additional 160 and 320 mg/kg of FA (OF+FA160 group and OF+FA320 group, respectively). *M. nipponense* (initial weight: 0.140 ± 0.015 g) were randomly divided into four groups with six replicates (60 individuals per replicate) and reared for a period of 10 weeks. The results showed that the OF treatments significantly reduced the growth performance, the expression of antioxidant genes in the hepatopancreas, the levels of low-density lipoprotein cholesterol, and the gene expression levels of *ACC*, *FAS*, *FABP10*, *ACBP*, *G6PDH*, and *SCD* in the hepatopancreas (*p* < 0.05). OF supplementation significantly increased the levels of high-density lipoprotein cholesterol in hemolymph and the gene expression levels of *CPT1* (*p* < 0.05). Addition of FA to the OF group significantly increased total bile acids (*p* < 0.05). In addition, it was found by Oil Red staining that the proportion of lipid droplets was significantly increased in the OF group (*p* < 0.05). However, the lipid droplets were alleviated by FA supplementation in the diet. OF was found to significantly reduce the diversity of intestinal microbiota by 16S rDNA sequencing and significantly increase the Firmicutes/Bacteroidetes (F/B) ratio (*p* < 0.05). Functional analysis of gut microbiota also showed that OF reduced lipolysis and led to fat deposition, which is related to gut microbiota. However, this study found that the composition of the gut microbiome of *M. nipponense* was changed by the addition of FA in the diet, including an increase in the abundance of *Ruminococcaceae UCG-005* and Lachnospiraceae, a reduction in the F/B ratio, and an improvement in lipid metabolism. In conclusion, the OF induced oxidative stress, disturbed the balance of intestinal microbiota, promoted lipid accumulation, and caused disorders of lipid metabolism in *M. nipponense* by increasing lipid synthesis and reducing β-oxidation. However, the results of this study highlighted the potential of FA supplementation to modulate intestinal microbial composition, promote bile acid production, and activate genes related to lipid metabolism in the hepatopancreas, ultimately leading to a reduction in lipid deposition in *M. nipponense*.

## 1. Introduction

Dietary lipids serve as the primary source of energy, provide vital nutrients such as fatty acids, fat-soluble vitamins, sterols, and phospholipids, and regulate animal health, development, reproduction, and organ functions [1]. Fish oil has attracted considerable attention as a primary lipid source for the development of commercial diets for aquatic organisms due to its richness in polyunsaturated fatty acids such as eicosapentaenoic acid (EPA) and docosahexaenoic acid (DHA) [2,3]. EPA and DHA are very easily oxidized during feed storage and processing, producing harmful chemicals such as aldehydes, ketones, and hydroperoxides [4]. The oxidation process produces oxidative by products that are easily absorbed and transported into the tissue, potentially triggering oxidative stress [1]. The addition of fish oil (OF) in the diet in largemouth bass after 12 weeks of rearing caused liver damage [5], growth suppression [6], and lipid deposition [7]. This ultimately leads to reduced growth and the occurrence of diseases. Nevertheless, there is limited research on the harmful effects of OF on crustaceans.

Given that the byproducts of fish oil oxidation have deleterious effects on aquatic organisms, the use of antioxidants could be a viable method to control lipid oxidation and reduce lipid accumulation. Nevertheless, few studies have been conducted on the incorporation of antioxidants into OF to mitigate the cytotoxic consequences. As a naturally occurring antioxidant, ferulic acid (FA) is mainly used in skin care and human health [8]. Due to its chemical composition, FA can effectively neutralize reactive molecules (e.g., hydroxyl, nitric oxide, and peroxides) and inhibit lipid peroxidation [9]. At the same time, the study has found that FA may prove to be a potential supplement and replacement nutrient that specifically targets acyl-CoA synthase 1 (ACSL1) and improves lipid metabolism in patients with diabetes [10]. In aquaculture, relevant studies have found that FA can reduce the oxidative stress of *Oreochromis mossambicus* when consuming OF, stabilize intestinal morphology, and improve digestive capacity and microbial community stability [11]. FA supplementation has also been shown to regulate bile acid (BA) secretion and reduce lipid deposition in grass carp (*Ctenopharyngodon Idellus*) [12]. 

The gut microbiome serves as a secondary metabolic organ of the host, primarily involved in the processing of carbohydrates, energy, fat, and amino acids by expressing over 100 times more host genes than its own [13]. However microbial dysbiosis in the intestine can lead to metabolic disorders within these microbial populations [14,15]. This can also significantly contribute to abnormal fat metabolism. There are many studies on OF in aquatic animals, for example, it negatively affected the diversity and structure of the gut microbiota of hybridized grouper (♀ *Epi-nephelus fuscoguttatus* × ♂ *Epinephelus lanceolatus*) [16], as well as significantly reduced the abundance of dominant microbiota in juvenile sea urchins (*Strongylocentrotus intermedius*) [17]. Therefore, the balance of the gut microbiota is crucial for maintaining the host’s well-being. Significantly, hazelnut oil supplementation effectively mitigated the deleterious effects of OF on the intestinal microbiota of tilapia, thereby ensuring the stability of the intestinal microbial community [6]. So far, there are fewer studies on the effects of OF and FA on the gut microbiota of aquatic crustaceans. 

*Macrobrachium nipponense* is a significant economical and nutritional freshwater crustacean and is widely cultivated in Asia [18]. China regards it as a vital crustacean species due to its robust adaptability, amazing fecundity, accelerated growth, and brief reproductive cycle [19]. Fish oil could be used as a main feed lipid source in *M. nipponense* [20]. However, the fish oil in the feed is prone to oxidation during the preparation and storage process [21]. This study investigated the influence of OF on the growth, oxidative stress, gut microbiome, and lipid metabolism of *M. nipponense*. In addition, this study aimed to analyze the potential of FA to mitigate the negative effects of OF.

## 2. Materials and Methods

### 2.1. Ethics Statement

The Animal Care and Use Committee of Nanjing Agricultural University endorsed this research (Nanjing, China), following strict adherence to national guidelines on laboratory animal care. The procedures adhered to institutional protocols at Nanjing Agricultural University. Animal welfare was prioritized above all else in accordance with FFRC-CAFS regulations (LAECFFRC-2021-06-30).

### 2.2. Oxidized Fish Oil Preparation

Fish oil was purchased at the market. Oxidized fish oil was prepared under laboratory conditions by adding Fe^2+^ (30 mg/kg, FeSO_4_·7H_2_O, Haikou Lvhengyuan Biotechnology Co., Ltd. Hainan, HI, CHN), Cu^2+^ (15 mg/kg, CuSO_4_·5H_2_O, Sinopharm Chemical Reagent Co., Ltd, Shanghai, SH, CHN), H_2_O_2_ (600 mg/kg, Shanghai Lingfeng Chemical Reagent Co., Ltd, Shanghai, SH, CHN), and water (0.3%) in proportion. After fully mixing, the fish oil was stirred at 37 ± 1 °C to produce oxidized fat with a certain degree of peroxide, according to our previous experiment [22]. After 14 days of oxidization, 150 mg/kg of ethoxyquinoline was added to prevent further fish oil oxidation. Ethoxyquinoline (S30950) was obtained from Shanghai Yuanye Bio-Technology Co., Ltd (Shanghai, SH, CHN). Its chemical name is 6-Ethoxy-2,2,4-trimethylquinoline, its purity is 90%, and its molecular formula is C_14_H_19_NO. The peroxide value (POV) was determined according to the Chinese national standard [23,24] (GB/T 5538-2005/ISO 3960:2001), and the POV of OF was 318 mmol/kg. A control POV of 2.2 mmol/kg for fresh fish oil was evaluated before feed formulation and stored at −20 °C to prevent further oxidation.

### 2.3. Experimental Diet

The formulation and proximate composition of the trial diets are shown in Table 1. The control diet contained 3% fresh fish oil (CT group), while the experimental diet contained 3% oxidized fish oil with an additional supplement of 0 (OF group), 160 mg/kg (OF+FA160 group), and 320 mg/kg FA (OF+FA320 group). Minor adjustments were made to the wheat flour content to balance the formulations. Various raw materials were crushed through a 60-mesh sieve and mixed gradually; then, oil and the appropriate amount of water were added. A twin-screw extruder (Guangzhou Huagong Optical Mechanical & Electrical Technology Co. Ltd. in Guangzhou, China) was utilized to granulate sinking pellets. After the feed was made in the form of granules of 1 nm particle size, it was naturally air-dried and dispensed, and subsequently stored in a −20 °C refrigerator. Ferulic acid (FA) with a purity of 99.1% was obtained from Guangzhou Cohoo Biotechnology Co., Ltd (Guangdong, GD, CHN). Crude protein and lipid content was measured by the Kjeldahl method (Kjeltec TM 8400, FOSS, Sweden) and by the Soxhlet method (Buchi 36,680, Switzerland). Lysine and methionine calculations were calculated according to the diet formulation table. Gross energy was calculated by using fuel values of protein, lipid, and carbohydrate. All dietary formulas were naturally air-dried. 

### 2.4. Prawns and Management

The prawns were from the Dapu breeding farm of the Freshwater Fisheries Research Center of the Chinese Academy of Fishery Sciences (Wuxi, China). Before experimentation, prawns were provided a common commercial feed. After 2 weeks of acclimatization, *M. nipponense* with similar size (0.140 ± 0.015 g) were randomly divided into 24 circular fiberglass tanks (φ1.5 m, 800 L water per tank) with 60 prawns in each tank, and the right amount of fake aquatic plants was added to reduce the fighting of the prawns in each tank. Prawns received manual feedings thrice a day at 7:30, 13:00, and 18:00. Residue was collected post-feeding, dried, and calculated. Feeding comprised 5–10% of their body mass. The water flow in the circulation system was maintained at 1 L/min. Rearing conditions included a temperature of 29–30 °C, a pH range of 7.0–7.5, and dissolved oxygen levels above 5 mg/L, with ammonia nitrogen and nitrite levels below 0.1 mg/L.

### 2.5. Sampling

After a feeding period of 10 weeks, total weight and dietary intake were measured to calculate the weight gain rate, specific growth rate, and feed conversion rate. Twelve prawns from each group (2 per tank, 6 replicates) were randomly selected for hemolymph collection and weight assessment. Hemolymph was obtained using Alsever’s solution (comprising 13.2 g/L trisodium citrate solution, 14.7 g/L glucose, and 4.8 g/L citric acid), mixed 1:1 with the sample, then centrifuged at 4000 rpm/min at 4 °C for 10 min. Hepatopancreas tissue (2 per tank, 12 per group) was preserved at −20 °C for antioxidant enzyme assessment. Another twelve hepatopancreases and intestines were stored at −80 °C for subsequent gene expression and gut microbiome analyses. Hepatopancreatic tissue was preserved in 4% paraformaldehyde, facilitating hepatic histology.

### 2.6. Growth Performance Parameters

The associated formulas are presented here:

Survival rate (SR, %) = 100 × final number of prawns/initial number of prawns;

Specific growth rate (SGR, %) = 100 × [Ln (average weight of the final prawns) − Ln (average weight of the initial shrimp)]/cultured days; 

Weight gain rate (WGR, %) = 100 × (average final body weight − average initial body weight)/average initial body weight;

Feed conversion ratio (FCR) = feed consumption/prawns weight gain;

Relative Feed intake (RFI, %/day) = 100 × total amount of the feed consumed × 2/[(initial body weight + final body weight) × days].

### 2.7. Biochemical Parameter Analysis of Hemolymph and Hepatopancreas

The triacylglycerol (TG), total cholesterol (TC), high-density lipoprotein cholesterol (HDL-C), and low-density lipoprotein cholesterol (LDL-C) levels of the hemolymph were analyzed utilizing the Mindray BS-400 automated biochemical analyzer (Shenzhen Mindray Bio-Medical Electronics Co., Ltd., Guangdong, DG, CHN).

HDL-C, LDL-C, TG, TC, total bile acid (TBA) in the hepatopancreas, malondialdehyde (MDA), and TBA in the hemolymph were determined per the manufacturer’s guidelines using the Nanjing Jiancheng kit. The kit models were TG (A110-1-1), TC (A111-1-1), HDL-C(A112-1-1), LDL-C(A113-1-1), TBA(E003-2-1), and MDA(A003-1-2).

### 2.8. Real-Time PCR Measurements

RNA extraction: twelve hepatopancreas samples per group were extracted utilizing the RNAiso Plus Kit (Takara Co., Ltd., Kyoto, Japan). RNA purity and concentration were measured using a NanoDrop (DN-1000, Thermo Scientific, Massachusetts, MA, USA), with only samples possessing OD 260/280 nm ratios within the range of 1.8–2.0 being chosen for subsequent cDNA synthesis. Following RNA standardization, an ExScriptTM RT-PCR kit was employed to generate cDNA from 500 ng DNase-treated RNA, as per the manufacturer’s guidelines (Takara Co. Ltd., Japan). The quantification of various genes, including acetyl CoA carboxylase (*ACC*), fatty acid synthase (*FAS*), glucose-6-phosphate dehydrogenase (*G6PDH*), acyl-CoA binding protein (*ACBP*), fatty acid-binding protein 10 (*FABP10*), stearoyl-CoA desaturase (*SCD*), carnitine palmitoyl-transferase 1 (*CPT1*), superoxide dismutase (*SOD*), catalase (*CAT*), glutathione peroxidase (*GPx*), heat shock proteins 60 (*HSP60*), and eukaryotic translation initiation factor (*EIF*), was performed using real-time PCR. The primers used for this analysis were custom-designed and obtained from GENCEFE Biotech Co. Ltd. (Wuxi, China), as previously reported by Zhao et al. in 2017. The assay was performed with the SYBR^®^Premix ExmTaq II (TliRNase Plus) Solution implemented on an ABI 7500 real-time PCR platform. Details about this methodology can be found in our earlier publication [25]. Expression levels were calculated utilizing the 2^−ΔΔCT^ metric. Table 2 shows the primer sequences involved and where they are cited, etc.

### 2.9. Histology Study 

Livers were preserved in 4% paraformaldehyde (PFA) at 4 °C and then bathed in 0.2 M phosphate buffer for 30 min twice with each wash [31]. In each group, three samples were randomly selected for sampling. Tissues were immersed sequentially in 0.2 M phosphate buffer containing 10%, 20%, and 30% sucrose for 3 h before cryosectioning. After briefly washing with sterilized water, sections were immersed in 60% isopropanol for 1 min and then stained with Oil Red O for 15 min. Hematoxylin was then applied before examination with a microscope camera.

### 2.10. 16S rDNA Sequencing and Gut Microbial Analysis

The microbial DNA was isolated from the samples using the E.Z.N.A.^®^ Soil DNA kit (Omega Bio-Tek, Norcross, GA, U.S.) following the manufacturer’s guidelines [32]. Three samples were randomly selected for sampling in each group. Following amplification at the V3-V4 region using 341F and 805R barcode fusion primers, the Illumina MiSeq platform facilitated high-throughput sequencing. The PCR-amplified products were subsequently quantified, amalgamated, and purified. The refined library was sequenced utilizing the MiSeq instrument (Illumina, San Diego, CA, USA). Both the barcode and linker sequences were excised and merged with the paired-end reads to form an extended segment. Subsequently, intermediates with an average quality score of less than 20 and a length of less than 100 nucleotides were eliminated. Primer sequences with discrepancies or ambiguous bases (Ns) greater than 5% were omitted from subsequent evaluations. Non-assimilable reads were completely excluded. Consequently, spliced paired-end sequences were synthesized using FLASH website [33]. Using VSEARCH 2.23.0, the chimera sequences were eliminated using a mixture of Denovo and Uchime methods [34]. Subsequently, the UPARSE software version 7.1 was employed to group the sequences into operational taxonomic units (OTUs) based on a 97% sequence match threshold [35]. Each OTU was classified via uclust, followed by a transformation of the raw OTU data into a uniform table utilizing the R 4.4.1package phyloseq, which subsequently removed low-abundance OTUs from further scrutiny.

By combining the above growth and biochemical indices, the intestines of the CT, OF, and OF+FA160 groups were selected for online 16S analysis and designated as the CT, OF, and OF.FA groups. The R package vegan was used for alpha diversity assessment including OTU abundance, species richness, Chao1 index, ACE index, Shannon index, and Simpson index, as well as for beta diversity exploration in measures such as principal component analysis (PCA) and similarity analysis (ANOSIM). Next, linear discriminant analysis (LDA) was applied using the microeco R package to hierarchically organize the 16S amplicon data into R3 objects. To delineate the individual taxonomic layers, a Kruskal–Wallis test (alpha threshold: 0.05) was performed, after which the LDA score was logarithmically transformed and set to 4.0.

### 2.11. Statistical Analysis

The Mantel test analysis between antioxidant gene expression levels, biochemical parameters, and lipid metabolism gene expression levels was performed on the Tutools platform (www.cloudtutu.com) from 20 November 2023 to 25 November 2023. The significance threshold was set at a *p*-value of < 0.05. Functional prediction, microbiological, and performance indicators were included in the correlation analysis, and the data were analyzed by Pearson analysis and then graphed using the Gephi 0.10.1 software. Subsequent heatmaps were also analyzed for significant differences using Tutools. One-way ANOVA and *t*-test were performed using the SPSS 21.0 software (Chicago, IL, USA) among the experimental groups, and the graphs were drawn using the Prism 8.0 software. Data outcomes are reported in terms of mean and standard error (mean ± SE). Significance thresholds was set at *p* < 0.05; *p* < 0.01; and *p* < 0.001 and they indicated statistical significance.

## 3. Results

### 3.1. Growth Performance

Table 3 shows the growth performance of prawns. Prawns in all dietary treatments survived about 78−83% during the trial. The FW, WGR, and SGR in the group of OF were significantly reduced compared to that of the control group (*p* < 0.05), while the SR and FCR between the groups were not significantly different (*p* > 0.05). However, in the OF+FA160 group, WGR, SGR, SR, RFI, and FCR were not affected compared with the control group or OF (*p* > 0.05). FW, WGR, and SGR were significantly lower in the OF+FA320 group than in the control group (*p* < 0.05) and were not significantly different from the other groups (*p* > 0.05).

### 3.2. Biochemical Parameter Analysis 

Table 4 shows the content of hepatopancreas and hemolymph HDL-C, LDL-C, TG, TC, and TBA. Compared with the OF group, the hepatopancreas and hemolymph TG, TC, and LDL-C of the OF+FA160 group were significantly reduced (*p* < 0.05), with significant elevation in HDL-C and TBA levels for the OF+FA160 group (*p* < 0.05). The hemolymph HDL-C and hepatopancreas and hemolymph TBA of the OF group were significantly lower than those of the control group (*p* < 0.05), but the hemolymph HDL-C and hepatopancreas and hemolymph TBA of the OF+FA160 and OF+FA320 groups were not significantly different to those of the control group (*p* > 0.05). Compared with the OF group, the hepatopancreas TBA of the OF+FA320 group was significantly increased (*p* < 0.05), and hemolymph HDL-C was not significantly different (*p* > 0.05). The MDA levels in the OF group were significantly higher than those in the OF+FA160 and OF+FA320 groups.

### 3.3. Lipid Metabolism and Antioxidant-Related Gene Expression

**Figure 1:**
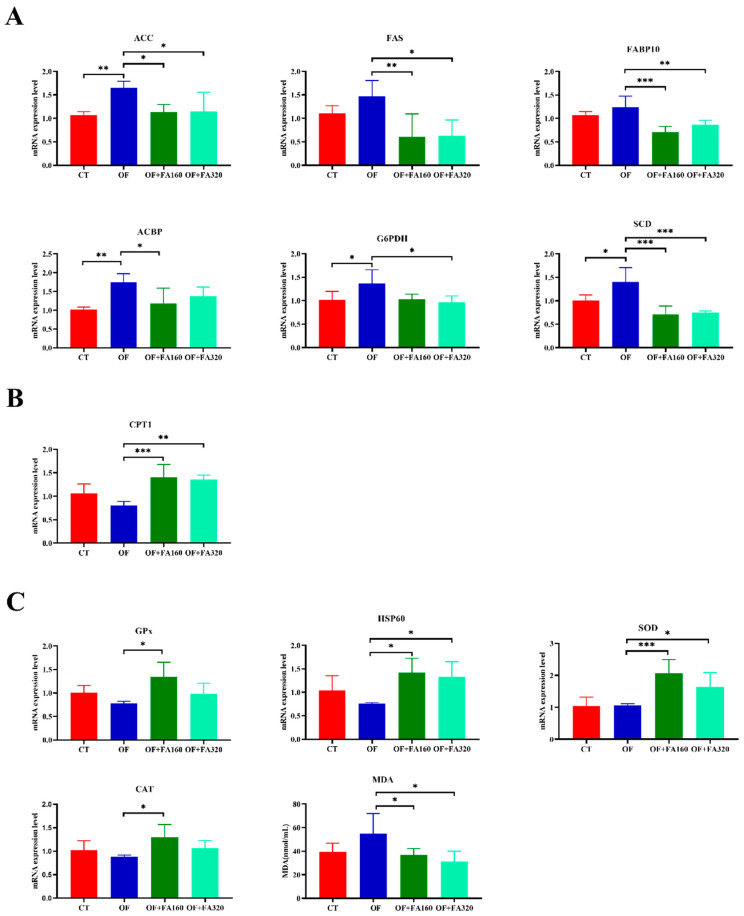
Effects of oxidized fish oil supplemented with ferulic acid on lipid synthesis, catabolism, antioxidant-related mRNA expression levels, and malondialdehyde (MDA) levels of *M. nipponense*. ACC: acetyl CoA carboxylase, FAS: fatty acid synthase, FABP10: fatty acid-binding protein 10, ACBP: acyl-CoA binding protein, G6PDH: glucose-6-phosphate dehydrogenase, SCD: stearoyl-CoA desaturase (**A**); CPT1: carnitine palmitoyl-transferase 1 (**B**); SOD: superoxide dismutase; HSP60: heat shock proteins 60, GPx: glutathione peroxidase, CAT: catalase (**C**). CT: control group; OF: 3% oxidized fish oil; FA: ferulic acid. Data are mean values of twelve replicates (2 per tank, 6 replicates) expressed as mean ± SE; *, *p* < 0.05; **, *p* < 0.01; and ***, *p* < 0.001 indicate significant differences.

Figure 1 shows the mRNA expression levels of the *EIF*, *ACC*, *CPT1*, *FAS*, *FABP10*, *G6PDH*, *ACBP*, and *SCD* genes. Compared to the OF group, the expression of the *ACC*, *FAS*, *FABP10*, *ACBP*, and *SCD* of the OF+FA160 group and the expression of the *ACC*, *CPT1*, *FAS*, *FABP10*, and *G6PDH* of the OF+FA320 group were significantly reduced, and the expression of the *ACC*, *G6PDH*, *ACBP*, and *SCD* of the CT group were also significantly decreased. The gene expressions of *CPT1*, *SOD*, and *HSP60* were substantially increased in the group of OF+FA160 (*p <* 0.001) and OF+FA320 (*p <* 0.01) compared with those of the OF group. Compared to the OF group, the expression of the *CAT* and *GPx* gene of the OF +FA160 group was significantly elevated (*p <* 0.05).

### 3.4. Oil Red O Staining 

**Figure 2:**
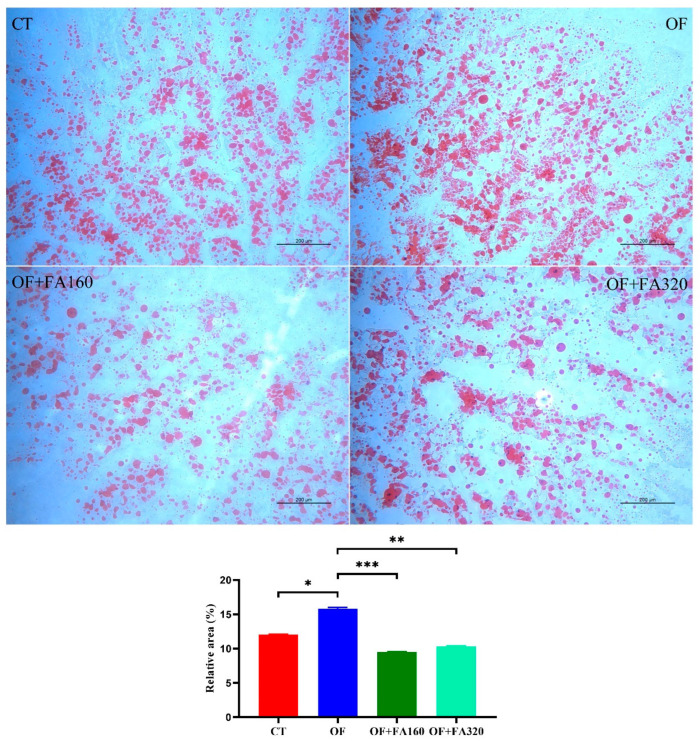
Effect of dietary oxidized fish oil with ferulic acid supplementation on Oil Red O staining micrographs of sections in the hepatopancreas of *M. nipponense* (magnification × 100). CT: control group; OF: 3% oxidized fish oil; FA: ferulic acid. Data are mean values of twelve replicates (2 per tank, 6 replicates) expressed as mean ± SE. Data are mean values of three replicates expressed as mean ± SE; *, *p* < 0.05; **, *p* < 0.01; ***, *p* < 0.001.

Figure 2 shows the results of Oil Red O staining. Compared with the CT group, the proportion of lipid droplets in the hepatopancreas of the OF group increased significantly (*p* < 0.05). The OF+FA160 and OF+FA320 groups significantly reduced the relative content of lipid droplets compared to the OF group (*p* < 0.05).

### 3.5. Diversity of Gut Microbiota

**Figure 3:**
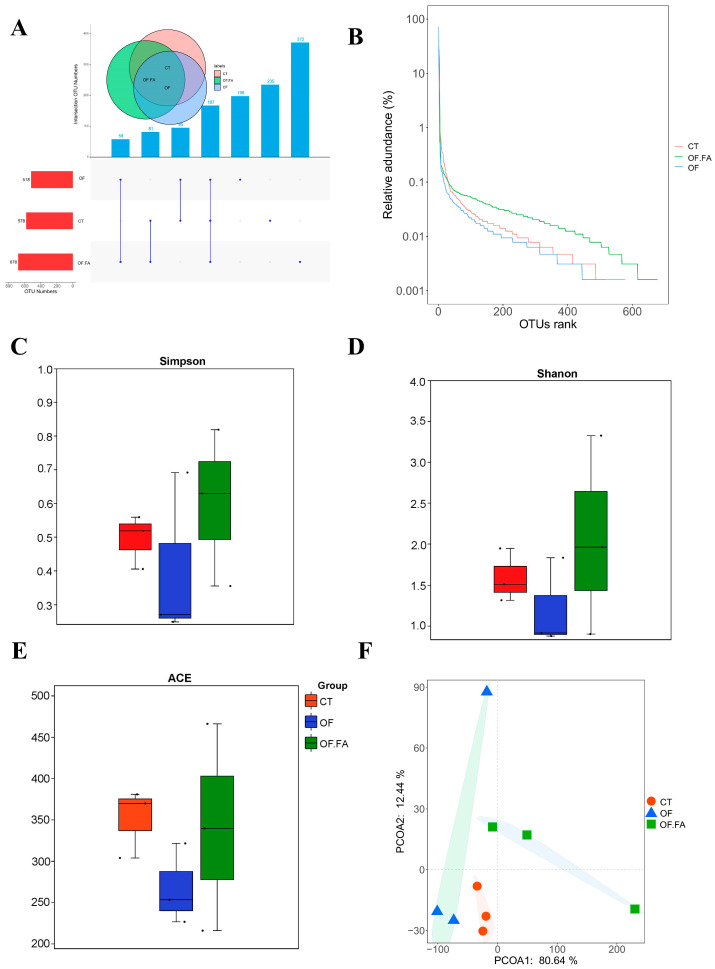
Upset diagram of gut microorganisms OTU number and α diversity and β diversity responses in the gut of *M. nipponense*. CT: control group; OF: 3% oxidized fish oil; FA: ferulic acid. Upset diagram showing the number of OTUs in the control, OF, and OF.FA groups (**A**), OTU rank curves (**B**), Simpson index (**C**), Shannon index (**D**), ACE index (**E**), and the principal co-ordinates analysis (PCoA) (**F**).

High-throughput 16S rDNA pyrosequencing revealed clustering of 97% similar effective reads into OTUs. Among the five treatment groups, including the CT, OF, and OF.FA groups, the OTU numbers were not significantly different (Figure 3A). However, the OTU rank curves, Shannon index, Simpson index, and ACE index analysis showed that the richness and alpha diversity of the colonic microbiota were significantly decreased in the OF group (Figure 3B–E). These indices exhibited an inconsistent trend; the control group stood out with statistical significance among all groups. The principal co-ordinates analysis presented a clear clustering of gut microbiota composition within groups for all groups, and the microbial composition differed between the groups (Figure 3F).

### 3.6. Composition of the Gut Microbial Community at Different Taxonomic Levels

**Figure 4:**
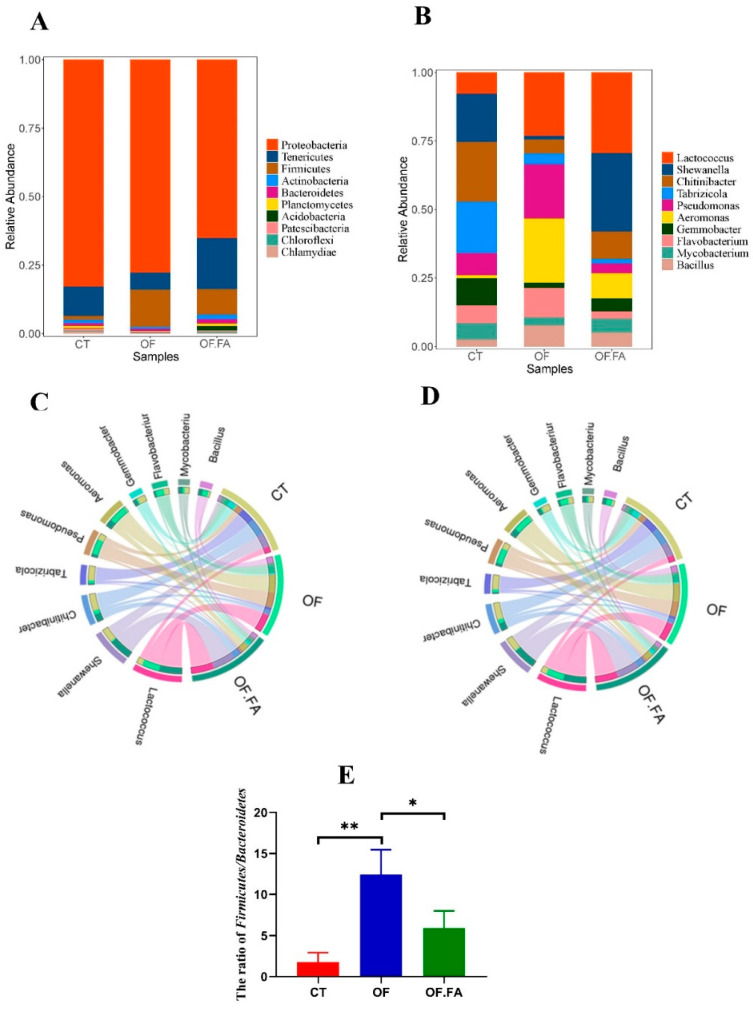
Effect of dietary oxidized fish oil with ferulic acid supplementation on the gut microbial composition of *M. nipponense*. CT: control group; OF: 3% oxidized fish oil; FA: ferulic acid. (**A**,**C**) Microbial composition in *M. nipponense* fed with different diets at the phylum level; (**B**,**D**) microbial composition in *M. nipponense* fed with different diets at the genus level; and (**E**) the ratio of Firmicutes/Bacteroidetes among groups. * *p* < 0.05, ** *p* < 0.01.

Circos diagrams delineated taxonomic profiles and species interactions within the microbiota. To evaluate the gut microbial taxa across three groups, CT, OF, and OF.FA, we assessed community composition variations at diverse taxonomic levels. The five most-dominant phyla were Proteobacteria, Tenericutes, Firmicutes, Bacteroidetes, and Actinobacteria (Figure 4A,C). Proteobacteria was the highest abundance phylum, accounting for 82.21 ± 3.27%, 77.22 ± 12.72%, and 63.77 ± 16.05% in the CT, OF, and OF.FA groups, respectively, followed by Tenericutes, accounting for 10.66%, 6.24%, and 18.30%, respectively. The following three phyla were Firmicutes, Bacteroidetes, and Actinobacteria, with respective proportions of 1.5%, 13.51%, 9.17%; 0.97%, 0.81%, 1.55%; and 1.19%, 0.63%, 1.58%.

At the genus level, the top 10 genera were identified in Figure 4B,D. The most dominant five genera were *Lactococcus*, *Shewanella*, *Chitinibacter*, *Aeromonas*, and *Pseudomonas*. In the control group, the most predominant genus was *Chitinibacter* (21.87%), followed by *Tabrizicola* (18.70%), *Shewanella* (17.59%), *Gemmobacter* (9.94%), and *Pseudomonas* (8.07%). *Aeromonas* (23.44%) was the most dominant genus in the OF group, followed by *Pseudomonas* (19.80%), *Lactococcus* (23.11%), *Flavobacterium* (10.67%), and *Bacillus* (7.67%). In the OF.FA group, the most predominant genera were *Lactococcus* (29.42%), *Shewanella* (28.61%), *Chitinibacter* (10.02%), *Aeromonas* (9.19%), and *Mycobacterium* (5.09%). There was a significantly elevated Firmicutes/Bacteroidetes (F/B) ratio in the OF group compared to the CT and OF.FA groups (*p* < 0.05) (Figure 4E).

### 3.7. Analysis of Microbial Composition of Different Groups

**Figure 5:**
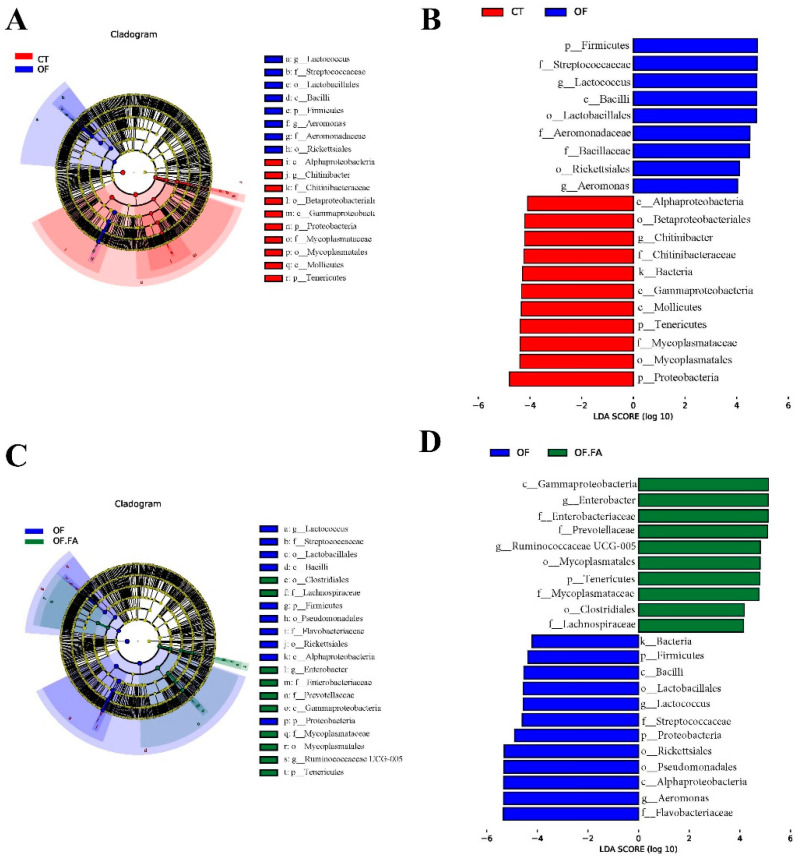
Linear discriminant analysis effect size (LEfSe) analysis. CT: control group; OF: 3% oxidized fish oil; FA: ferulic acid. (**A**,**B**): Cladogram of differential species and histogram of differential species between the CT group and OF group, respectively. (**C**,**D**): LEfSe analysis identified the most differentially abundant taxa between OF and OF.FA.

As exhibited in Figure 5, the linear discriminant analysis effect size (LEfSe) method was employed to analyze the gut microbiome. The differential OTUs from phylum to genus level in the OF group were compared to the control group, and the biomarkers were found to be Firmicutes, Streptococ-caceae, Lactococcus, Bacilli, Lactobacillales, Aeromonadaceae, Bacillaceae, Rickettsiales, and *Aeromonas* respectively. 

Combined with the results of the distribution histogram of differential OTUs (LDA score log10 = 4.0), the differential OTUs were classified into Alphaproteobacteria, Betaproteobacteriales, *Chitinibacter*, Chitinibacteraceae, Bacteria, Gammaproteobacteria, Mollicutes, Tenericutes, Mycoplasmataceae, Mycoplasmatales, and Proteobacteria between the control group and OF group (Figure 5A,B). The mean abundance exhibited an ascending trajectory post-FA addition, further demonstrating that the OF.FA dietary strategy noticeably elevated all taxonomic levels in Gammaproteobacteria, *Enterobacter*, Enterobacteriaceae, Prevotellaceae, *Ruminococcaceae UCG_005*, Mycoplasmatales, Tenericutes, Mycoplasmataceae, Clostridiales, and Lachnospiraceae (*p* < 0.05). Compared to the control group, Firmicutes, Bacilli, Lactobacillales, *Lactococcus*, Streptococcaceae, Proteobacteria, Rickettsiales, Pseudomonadales, Alphaproteobacteria, *Aeromonas*, and Flavobacteriaceae were biomarkers in the OF group (Figure 5C,D).

### 3.8. Functional Prediction of Gut Microbial Community

**Figure 6:**
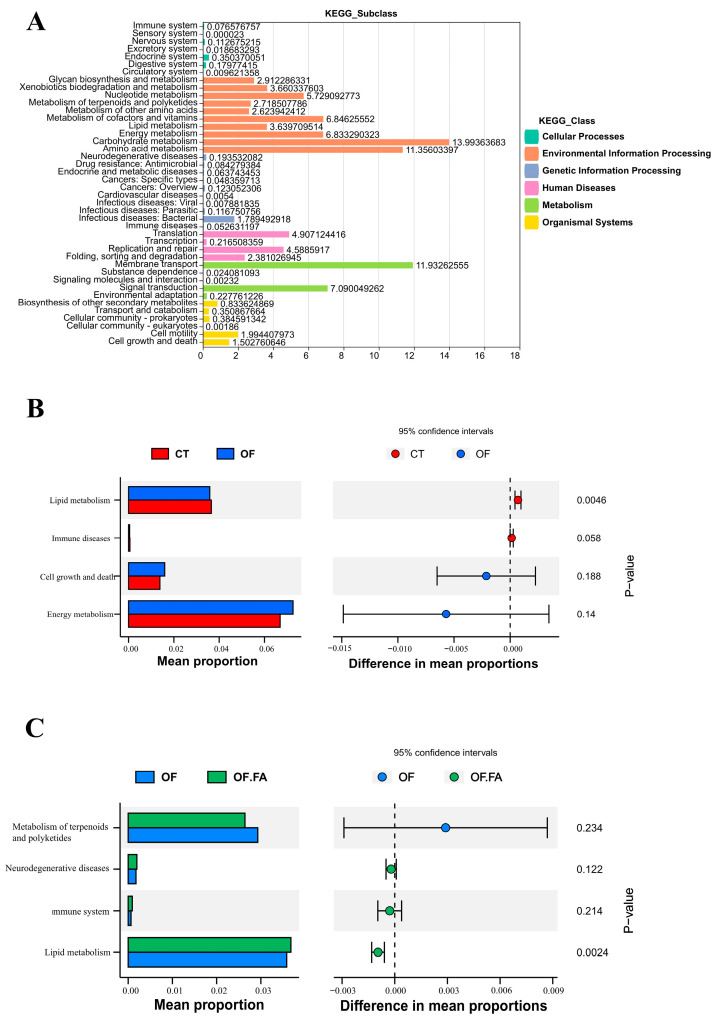

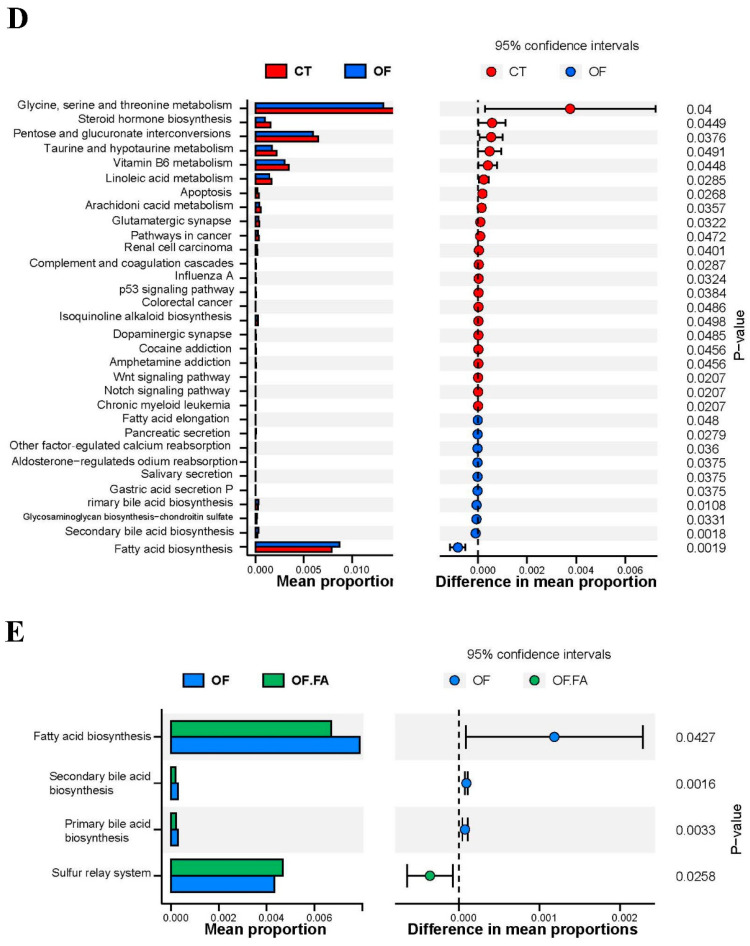
Effect of dietary oxidized fish oil with ferulic acid supplementation on gut microbiota function of *M. nipponense*. CT: control group; OF: 3% oxidized fish oil; FA: ferulic acid. (**A**): Classification of level 2 functional forecasts and their proportion of level 1 functional forecasts; (**B**,**C**): functional forecasts analysis on level 2 between CT and OF groups, as well as between the OF and OF.FA groups; (**D**,**E**): functional forecasts analysis between CT and OF groups, as well as between the OF and OF.FA groups at level 3. The middle showed the difference between the proportions of functional abundance in the 95% confidence interval (*p* < 0.05). The value at the rightmost part of the figure is the *p*-value. *p* < 0.05 represents a significant difference.

As shown in Figure 6, the control and therapeutic groups were assigned to six KEGG level 1 classifications: “Cellular Processes”, “Genetic Information Processing”, “Environmental Information Processing”, “Human Diseases”, “Metabolism”, and “Organismal Systems”, and the metabolism was predominant the KEGG pathway in all treatment groups (65.79%), including lipid, amino acid, and energy metabolism (Figure 6A). Compared with the OF group, a significant increase in lipid metabolism abundance was seen in the CT and OF.FA groups at KEGG level 2, (*p* < 0.05) (Figure 6B,C). Examining the gut microbial community of *M. nipponense* revealed distinct KEGG tertiary metabolic pathways. Compared to the CT group, the OF group activated fatty acid elongation, fatty acid biosynthesis, primary bile acid biosynthesis, and secondary bile acid biosynthesis (*p* < 0.05), but inhibited glycine, serine, and threonine metabolism, steroid hormone biosynthesis, pentose and glucuronate interconversions, taurine and hypotaurine metabolism, vitamin B6 metabolism, linoleic acid metabolism, apoptosis, arachidoni cacid metabolism, and the glutamatergic synapse (*p* < 0.05). Compared to the OF group, fatty acid biosynthesis, primary bile acid biosynthesis, and secondary bile acid biosynthesis were inhibited in the OF.FA group. Only the sulfur relay system was significantly activated (*p* < 0.05) (Figure 6D,E).

### 3.9. Gut Microbiota and Phenotypic Indicators Correlation Analysis

**Figure 7:**
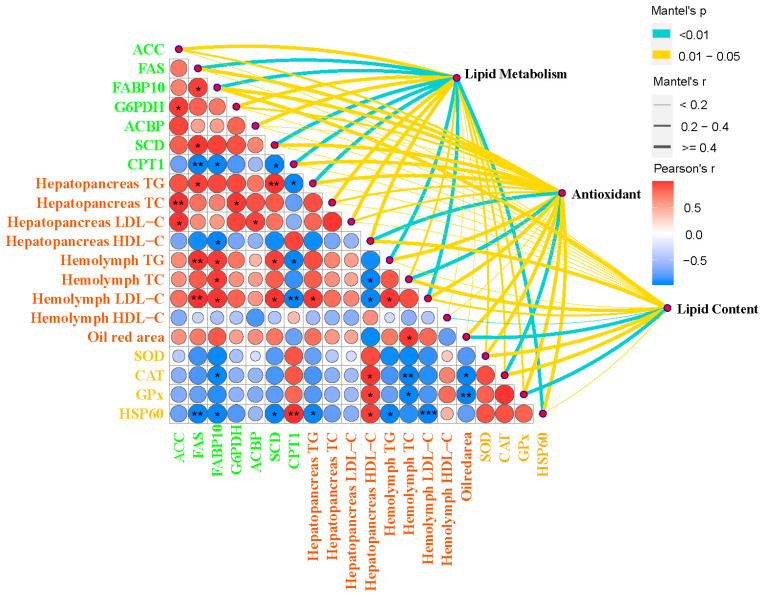
Multi-dimensional correlation heat map of gut microbes and phenotypes. The phenotypes were compared in pairs with each other, with a color gradient and color block size denoting Pearson’s correlation coefficients. Antioxidant capacity and lipid metabolism were related to phenotypes by partial Mantel’s tests. Edge width corresponds to Mantel’s r statistic for the corresponding distance correlations and edge color denotes the statistical significance based on 9999 permutations. * *p* < 0.05, ** *p* < 0.01, *** *p* < 0.001.

The analysis presented in Figure 7 explores the association between antioxidant capacity and lipid metabolism. The hepatopancreas TG was negatively correlated with *FAS* (*p* < 0.05) and *SCD* (*p* < 0.01). Additionally, the TC in the hepatopancreas was positively correlated with *ACC* (*p* < 0.01) and *G6PDH* (*p* < 0.05). The *ACC* and *SCD* were negatively correlated with hepatopancreas LDL-C (*p* < 0.05). The hemolymph TG and hemolymph LDL-C were positively correlated with *FAS* (*p* < 0.01), *FABP10* (*p* < 0.05), and *SCD* (*p* < 0.05) and were negatively correlated with *CPT1* (*p* < 0.05). The Oil Red area was positively correlated with hemolymph TC (*p* < 0.05) and was negatively correlated with hemolymph *CAT* (*p* < 0.05) and *GPx* (*p* < 0.05).

**Figure 8:**
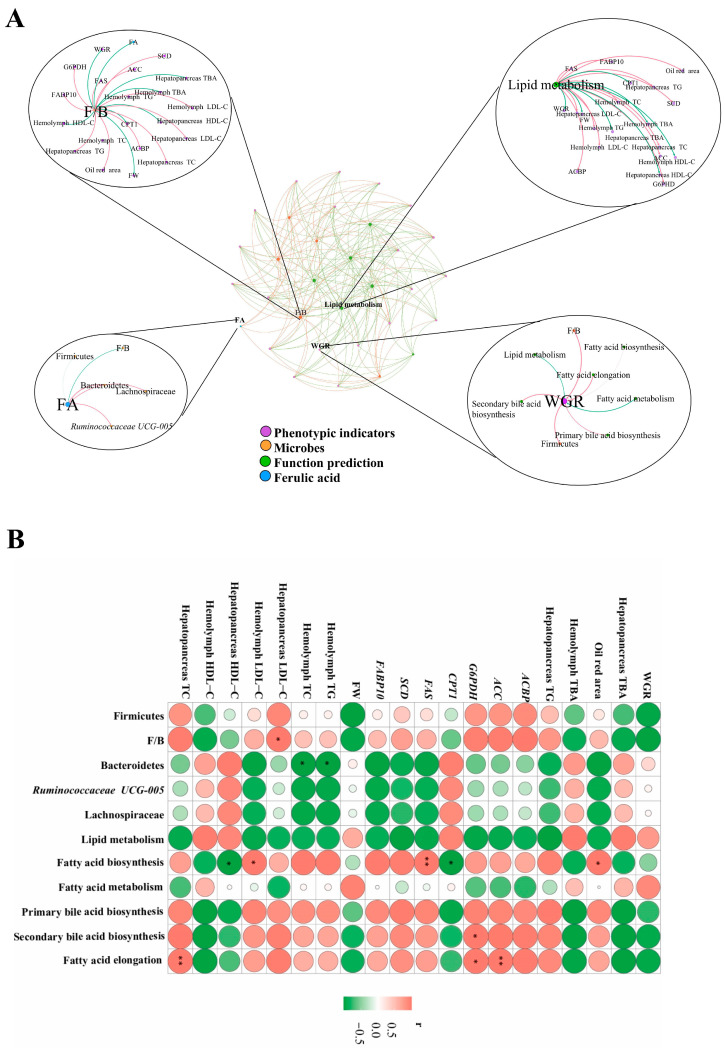
Correlation analysis of FA, microbes, function prediction, and phenotypic indicators in *M. nipponense* fed ferulic acid with oxidized fish oil. Note: (**A**): Correlation network analysis of FA, microbes, function prediction, and phenotypic indicators. The colors of the nodes indicate data types, and the colors of the lines indicate positive (red) or negative (green) correlation relationships. (**B**): Heatmap of Pearson’s correlations between phenotypic indicators, microbes, and function prediction. The colors range from green (negative correlations) to orange (positive correlations). Significant correlations are noted by * *p* < 0.05, and ** *p* < 0.01.WGR: weight gain rate; ACBP: acyl-CoA binding protein; ACC: acetyl CoA carboxylase; G6PDH: glucose-6-phosphate dehydrogenase; CPT1: carnitine palmitoyl-transferase 1; FAS: fatty acid synthase; SCD: stearoyl-CoA desaturase; FW: final weight; F/B: Firmicutes/Bacteroidetes.

The correlation analysis of microbes, functional prediction, and phenotypic indicators are presented in Figure 8. The hepatopancreas LDL-C was positively correlated with *F/B* rate (*p* < 0.05). *Bacteroidetes* was negatively correlated with hemolymph TG and TC (*p* < 0.05). Fatty acid synthesis was positively correlated with *FAS* (*p* < 0.01), Oil Red area, and hemolymph LDL-C (*p* < 0.05), and was also negatively correlated with *CPT1* and hepatopancreas HDL-C (*p* < 0.05). Fatty acid elongation was positively correlated with *G6PDH* (*p* < 0.05) and *ACC*, hepatopancreas TC (*p* < 0.01). FA showed a positive correlation with Bacteroidetes, Lachnospiraceae, and *Ruminococcaceae UCG-005*, and a negative correlation with F/B.

## 4. Discussion

Numerous studies have indicated a decrease in the growth and feed efficiency of aquatic animals when fed with OF [36], which is generally consistent with the present study. A previous study showed that consumption of OF disrupted the lipid metabolism of aquatic animals [7]. The reduction in growth performance can be attributed to the reduction in nutritional value due to oxidation of HUFA in the diet and oxidative stress due to the toxic peroxidation products of OF [36]. However, after adding a proper amount of FA, it was found that the growth performance was significantly improved, which was similar to the relevant research results [11]. The beneficial effect could be due to the antioxidant abilities of FA, which protect against lipid peroxidation and thus ensure the normal growth of aquatic animals fed with the OF. However, based on the results, we found that the growth performance of the OF+FA320 group was not significantly different from that of the OF and OF+FA160 groups; this may be since 320 mg/kg FA exceeds the optimal level of addition, which was also found in a previous study on FA in *M. nipponense*, where a regression analysis of the growth performance yielded an optimal level of FA supplementation of 160 mg/kg [37], and 320 mg/kg FA could alleviate a certain degree of oxidative stress but had no direct effect on growth performance.

The hemolymph biochemical parameters of aquatic animals species were crucial components in assessing the nutritional and health status of the organism [38]. The concentration of TCHO and TG serves as a crucial indicator suggesting modifications in lipid metabolism [39]. LDL-C and HDL-C are important lipoproteins that contain abundant cholesterol; LDL-C is used to transport cholesterol molecules from the liver throughout the body. Excess amounts of oxidized low-density lipoproteins trigger the accumulation of cholesterol in arterial walls, leading to arterial hardening, while HDL-C facilitates the movement of cholesterol from the surrounding tissues to the liver, ultimately promoting cholesterol metabolism [40]. The results of this study showed that after adding OF to the diet, TG, TC, and LDL-C increased in the hemolymph of the OF group, but after adding 160 mg/kg FA to the diet of OF, the TG, TC, and LDL-C decreased significantly, while HDL-C showed the opposite results. This result may be attributed to the fact that OF leads to lipid metabolism disorders, which can be alleviated by adding a certain amount of FA. FA can scavenge some of the lipid peroxides produced by OF and alleviate lipid metabolism disorders by regulating the expression of enzymes and inducing the expression of these antioxidant enzymes. As a result, there is an increase in TG, TC, and LDL-C levels, while HDL-C levels in the hemolymph decrease. These results are consistent with a previous study on tilapia (*O. mossambicus*) by Yu [11] and suggest that lipid metabolism disorders may be present in the oxidized fish oil-treated groups. In the OF+FA160 group, a significantly increased reduction in LDL-C and an increase in HDL-C was observed, underlining the positive influence of FA on lipid metabolism. The accumulation of certain bile acids in the liver that have hepatotoxic effects is balanced by others that have protective properties against liver lipid deposits and inflammation [41]. In addition to suppressing the biosynthesis of fatty acids and triglycerides, bile acids can also activate the elimination of triglycerides from serum by stimulating lipoprotein lipase and causing the hydrolysis of triglycerides in VLDL and chylomicrons [42]. Our study found that the levels of hemolymph and hepatopancreatic TBA were noticeably higher in the OF+FA160 group compared to the OF group. Considering the lipid content of the samples, these results suggest that FA reduces lipid accumulation by increasing TBA concentration.

To explore the mechanism underlying oxidative stress and its impact on lipid metabolism and to determine whether the inclusion of the natural antioxidant FA can alleviate this effect, this study focused on examining genes associated with antioxidant activity, fatty acid production, and fatty acid β-oxidation. The antioxidant defense of crustaceans predominantly utilizes SOD, CAT, and GSH-dependent GPx enzymes [43]. SOD and CAT initially counteract oxidative stress, intercepting free radicals and safeguarding biomolecules, while GPx is a crucial antioxidant enzyme in crustaceans. These antioxidant genes efficiently eradicated reactive oxygen species like superoxide anions and hydrogen peroxide in the organism [44,45]. The results showed that the expression of *SOD*, *CAT*, *GPx*, and *HSP60* in the OF group was lower than those in the CT group, suggesting that the addition of OF could lead to oxidative stress, which is consistent with the results of the group that showed a similar attractiveness of the antioxidant enzyme activity of hemolymph. Similar results have been found in sterlet (*Huso huso* ♂ × *Acipenser ruthenus* ♀) [7]. In contrast, the expressions of *SOD*, *CAT*, and *GPx* were significantly higher in the OF+FA160 group compared to the OF group. Additionally, studies have shown that FA has antioxidant properties that mitigate oxidative stress by reducing the formation of lipid peroxides. This minimizes damage caused by reactive oxygen [46].

There may be a potential correlation between lipid deposition and oxidative stress. Traditional Oil Red O staining employed for detecting triacylglycerols, cholesteryl esters, and steatosis is a valuable tool for histological diagnosis [47]. When analyzing the proportion of lipid droplets stained with Oil Red O in our research, we discovered a significant increase in their size within the OF group compared to both the CT and OF+FA160 groups. Because lipid droplets are composed of TG and sterol esters [48], cells use lipids as a reservoir for metabolic energy and precursors for membrane components that are stored in organelles called lipid droplets [49]. It was clear from the results of the study that OF in the diet led to oxidative stress, which may lead to disruptions in lipid metabolism and subsequent lipogenesis. Finally, lipid deposits occurred in the hepatopancreas. Other studies on OF found that it increased lipogenesis and suppressed lipolysis, induced oxidative stress via reduced GPx activity, increased malondialdehyde (MDA) content, and damaged mitochondrial structure in sterlet [7]. Oil Red O staining showed that dietary FA reduced the accumulation of lipid droplets in the hepatopancreas. This correlated positively with the results of the hemolymph and hepatopancreatic TG, TC, HDL-C, and LDL-C study. Combined with the results of the expression of antioxidant genes, it can be found that FA could alleviate oxidative stress, promote lipid metabolism, and reduce lipid deposition. This is consistent with Cho’s study, which demonstrated that ferulic acid exhibited weight loss and improved glucose homeostasis, lipid profiling, and hepatic steatosis in a HFD-induced mouse model [50], and similar results were found at the cellular level by Koh [51].

Firstly, FAS and ACC served as multiple enzyme composites and represented animal lipid metabolic biomarkers with regulatory roles in de novo fatty acid synthesis [52]. Hepatopancreas FAS and ACC demonstrated a vital role in lipogenesis. The current study found that dietary OF upregulates these genes, increases fatty acid synthesis, and induces lipid deposition in the hepatopancreas. ACBP combines with acetyl-CoA for β-oxidation, generates phospholipids and TG, and produces ATP [53]. Fatty acid binding proteins (FABPs) are involved in the intracellular uptake and transport of fatty acids, which bind fatty acids and transfer them to their binding sites [54]. CPT1 was a key rate-limiting enzyme of fatty acid β-oxidation [55]. We found that *CPT1* and *ACBP* were downregulated in the OF group, which could cause the fatty acid oxidation level of *M. nipponense* to be lower in the OF group. There is also the possibility that *ACC* was upregulated in the OF group, which may inhibit the expression of *CPT1*. Similar results were found in the high-dose (HD) diets in Chen’s study [56]. It has been speculated that oxidative stress caused by OF may be the result. Analyzed at the molecular level, OF caused the disruption of lipid metabolism by upregulating key genes controlling de novo fatty acid synthesis and repressing genes encoding critical rate-limiting fatty acid β-oxidation enzymes. Consequently, it stimulates lipid biosynthesis and suppresses fatty acid catabolism, which may lead to increased intracellular TG levels. Traditionally, G6PDH has been identified as a source of NADPH for lipid production [57]. G6PDH’s role as a catalyst in the pentose phosphate (PPP) pathway provides substantial cellular NADPH required for fatty acid biosynthesis, as witnessed in Wu et al.‘s research [58]. Here, the increased expression of *G6PDH* in the OF group resulted in increased enzyme activity and increased NADPH generation, which produces large amounts of fatty acids for fat synthesis. This promotes lipid accumulation in *M. nipponense*. Significant down-regulation of *FAS*, *ACC*, *G6PDH*, and *ACBP* expression and a significant increase in *CPT1* were found in the OF+FA160 and OF+FA320 groups, and the extreme value was reached in the OF+FA160 group. The results suggest that FA can alleviate the oxidative stress caused by OF and the resulting lipid deposition. A related study confirmed the role of oxidative stress in obesity, and N-acetylcysteine (NAC), an antioxidant, significantly reduced ROS-induced lipid accumulation [59], which was similar to the present study. 

Previous studies confirm the status of the gut microbiome as a “host organ” or secondary genome. These studies demonstrate that complex microbial compositions promote host health [60]. Various research findings have uncovered a remarkable decline in both species count and species richness along with a decline in gut microbial diversity after consuming an OF regimen [16]. In our study, we also found that OF was found to curtail alpha diversity in gut microbiota, this is manifested by the observed species’ Simpson, Shannon, and ACE indices, which showed a decreasing trend after the addition of OF in diets. Compared with the OF group, OF diet with FA supplementation could significantly improve the number of species and species richness. FA has also been found to increase the number of species and species richness of gut microbiota in the gut of zebrafish (*Danio rerio*) [61]. The microbial population similarities among the control group, OF group, and OF.FA group were compared by PCoA analysis and ANOSIM analysis. The results exhibited that the microbial composition has differences among groups. The results are similar to previous studies suggesting that diet affects gut microbial diversity [62]. 

The gut microbiome significantly influences the host’s energy balance as well as glucose and lipid metabolism [63]. In this study, Proteobacteria dominated within the gut microbiota of *M. nipponense*, followed by Firmicutes, Tenericutes, Bacteroidetes, and Actinobacteria. Each phylum of gut microbiota will have unwanted effects on the host. This research discovered significant alterations in the gut microbiome of *M. nipponense* related to dietary variations. Relevant studies have indicated that the Firmicutes species may increase hepatic steatosis by affecting fatty acid influx and lipogenesis [64]. The addition of OF to the diet led to an increase in the abundance of Firmicutes, while Bacteroidetes showed a decreasing trend, and the addition of FA on top of it resulted in the exact opposite trend, as found in this study. In the correlation in Figure 8, it was found that Bacteroidetes showed the opposite trend to hemolymph TG and TC. Combined with the correlation analysis of fat content and lipid metabolism gene expression levels, we hypothesized that OF would lead to an increase in the abundance of Firmicutes and promote fatty acid synthesis as well as lipid deposition, while the addition of FA would regulate the intestinal bacterial microbiota and promote lipid metabolism, to achieve the effect of lowering lipids and promoting the health of the gut. Previous studies have shown that an alteration in the Firmicutes/Bacteroidetes (F/B) ratio is a hallmark of gut microbiota dysbiosis in obesity [65]. In this study, a significant increase in F/B was found after the addition of OF to the diet and a significant decrease in this value was found after the subsequent addition of 160 mg/kg FA, and a positive correlation was found between F/B and hepatopancreatic LDL-C (Figure 8). The results showed that FA enhanced bacterial diversity, diminished the F/B ratio, and rectified gut dysbiosis instigated by OF diet. This was similar to the finding that resveratrol butyrate esters (RBEs) improved lipid metabolism [66] and citrus limon (lemon) can reduce lipid levels and improve liver function [67]. 

*Ruminococcaceae UCG-005* were more abundant in the OF.FA group than in the OF group in the present study by LEfSe analysis. Previous research identified *Ruminococcaceae* as cellulose-degrading bacteria that produce SCFAs [68] and found that *Ruminococcaceae UCG-005* was negatively correlated with body weight and obesity-linked metabolic indicators [69]. Therefore, we hypothesized that FA promoted host lipid metabolism by increasing the abundance of *Ruminococcaceae UCG-005*. We found significantly increased abundance of Lachnospiraceae in the OF.FA group compared to the OF group by linear discriminant analysis (LDA) effect size (LEfSe) results, which is similar to the recent findings in lipid-lowering studies that supplementation with BGP-Z31 can effectively reshape gut microbiota structure and reduce lipids by increasing *Bacteroides*, Muribaculaceae, and Lachnospiraceae and reducing F/B ratio and the *Desulfovibrionaceae* count [70]. 

The intestinal microbiome is an essential component in maintaining the well-being of its host. Changes in the bacterial balance of the intestine can lead to the development of metabolic disorders [71]. Predicting the health status of the gut microbiota is a promising but underdeveloped area. In this study, features were grouped and analyzed. Through KEGG enrichment analysis, level 1 functional predictions are typically categorized into six broad categories [72], which are similar to the results of the appellate functional predictions. Nutrient metabolism is one of the important functions of intestinal microbiota [73], especially lipid metabolism. The KEGG level 2 functional predictions relative abundance in the gut microbiota suggested that the addition of OF to the diet led to a significant decrease in the functional abundance of lipid metabolism compared to the control and OF.FA groups, which also suggests that oxidized fish oil disrupted the lipid metabolism of the shrimp gut microbiome, while FA replenishment improved microbiota balance and restored functionality. Other studies have also found that FA therapy significantly alleviated diabetes-related metabolic disorders in mice [10]. Further analysis of the KEGG level 3 function of the intestinal microbiota revealed that in the OF group, the abundance of fatty acid synthesis and fatty acid elongation were significantly higher in the CT and OF.FA groups, which is consistent with the results of the correlation analysis. In fish studies, OF was also found to induce fat accumulation by promoting fatty acid synthesis and inhibiting fatty acid oxidation [74]. Interestingly, previous studies have also found that FA downregulated fatty acid synthesis-related genes and decreased lipid deposition [61]. Bile acid production and lipid metabolism can be explored through metabolites produced by gut microbes. The gut microbiota and its metabolites have been found to influence lipid metabolism and liver health in studies on the addition of lithocholic acid to grass carp [75]. The present study suggested that FA improves gut microbiota function and reduces oxidized fish oil-induced lipid deposition reduction. In functional prediction, it was found that the abundance of primary bile acid and secondary bile acid synthesis was also significantly higher. The predictions of this function were opposite to the results for total bile acid content, and OF might lead to negative feedback regulation of total bile acid content in the hemolymph and hepatopancreas by the intestinal microbiota.

## 5. Conclusions

**Figure 9:**
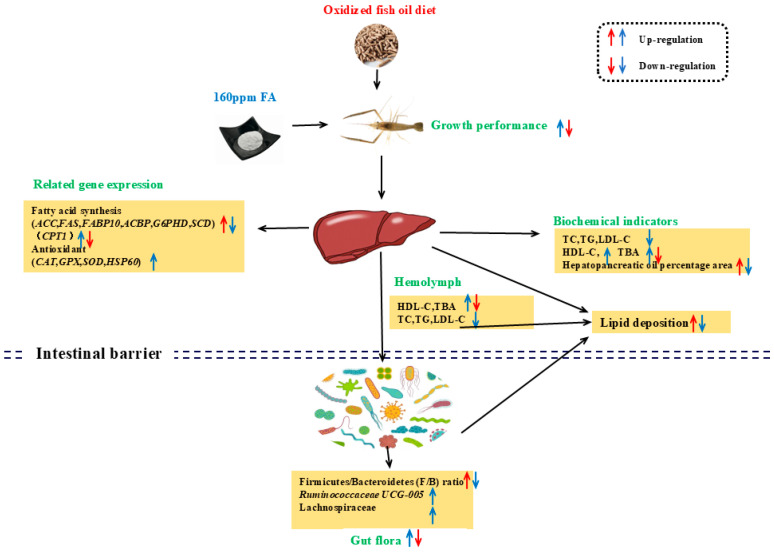
Flowchart summarizing the experiment.

In summary, our data indicated that dietary OF increased oxidative stress, reduced the diversity of intestinal microbiota, and caused disorder in the structure of intestinal microbiota, which might bring out disorders of lipid metabolism by upregulating key genes regulating de novo fatty acid synthesis and downregulating key rate-limiting enzymes of fatty acid β-oxidation genes, causing lipid deposits and lowering the growth performance. Supplementation of 160 mg/kg FA could minimize the oxidative damage induced by OF in *M. nipponense*, altering the gut microbiome primarily through reducing the F/B ratio and escalating the proportion of abundance of microbial communities, notably *Ruminococcaceae UCG-005* and Lachnospiraceae. In addition, 160 mg/kg FA could promote bile acid production and lipid metabolism and improve growth performance (Figure 9). This study provides a theoretical basis for the mechanism of using FA to reduce the negative effects of dietary oxidative stress on *M. nipponense*.

## Figures and Tables

**Table 1:** Ingredients and proximate chemical composition of the experimental diets (air-dried).

Ingredients (%)	CT	OF	OF+FA160	OF+FA320
Fish meal ^a^	30.00	30.00	30.00	30.00
Soybean meal ^a^	22.00	22.00	22.00	22.00
Rapeseed meal ^a^	10.00	10.00	10.00	10.00
Shrimp meal ^a^	6.00	6.00	6.00	6.00
Squid paste ^a^	3.00	3.00	3.00	3.00
Wheat flour ^a^	20.93	20.93	20.91	20.90
Fish oil ^b^	3.00	0	0	0
Oxidized fish oil ^b^	0	3.00	3.00	3.00
Soybean phospholipids ^b^	1.00	1.00	1.00	1.00
Ca(H_2_PO_4_)_2_^c^	2.00	2.00	2.00	2.00
Premix ^c^	1.00	1.00	1.00	1.00
Vitamin C ^c^	0.50	0.50	0.50	0.50
Choline chloride (60%) ^c^	0.50	0.50	0.50	0.50
Ecdysone (10%) ^c^	0.02	0.02	0.02	0.02
DMPT ^d^	0.05	0.05	0.05	0.05
Ferulic acid (99.1%) ^d^	0.00	0.00	0.016	0.032
Total	100.00	100.00	100.00	100.00
Proximate Composition (%, air-dried)				
Dry matter	89.36	89.47	89.43	89.53
Crude protein	40.65	40.62	40.58	40.56
Ether extract	8.24	8.21	8.25	8.24
Lysine	2.64	2.62	2.63	2.61
Methionine	0.92	0.91	0.89	0.93
Gross energy (MJ/kg)	17.09	17.01	17.12	17.10

Note: all dietary formulas are naturally air dried. CT: control group; OF: oxidized fish oil; FA: ferulic acid. ^a^ Provided by Dabeinong Co., Ltd. (Huaian, China). ^b^ Provided by Hulunbeier Sanyuan Milk Co., Ltd., Inner Mongolia, China. ^c^ Provided by Wuxi Hanove Animal Health Products Co., Ltd. (Wuxi, China). ^d^ DMPT(Dimethyl-beta-propiothetin) was Provided by Guangzhou Coho Biotechnology Co., Ltd. (Guangzhou, China).

**Table 2:** Real-time PCR sequence.

Gene	Primer sequences (5′–3′)	Product Length (bp)	Tm (°C)	Accession Number	Reference
*ACC*	(F) CAAGGTCCACTACATGGTCT (R) ACTCTTCCCAAACTCTCTCC	154	56.55	KP690138.1	(Luo et al., 2018) [26]
			56.17		
*FAS*	(F) CGGTCAGACAAACTACGGCT (R) CACTGAATAGCCACCCCAGG	93	60.04	MK307767.1	(Zhou et al., 2020) [27]
			60.11		
*CPT1*	(F) AATTTTTGACTGGCTTCTCC (R) TCCATTCTGGAAATCATCTG	176	54.06	KP690136.1	(Luo et al., 2018) [26]
			52.62		
*G6PDH*	(F) CGTGGACCTTTCTTCATTAG (R) ACCATCAACCATTTGAGAAG	164	53.75	KP690144.1	(Luo et al., 2018) [26]
			53.46		
*ACBP*	(F) GAGGCTGCTGAGAAGGTC (R) ATCATACCAGGTCGCTCC	122	57.07	KF896234.1	(Li et al., 2021) [28]
			55.74		
*FABP10*	(F) CCAAGCCAACTCTGGAAGTC (R) GATCTCAACGCTGGCTTCTC	218	58.47	JN995589.1	(Li et al., 2021) [28]
			58.71		
*SCD*	(F) ATAATGTTTGCCCTGCTACA (R) ATGTCATTCTGGAAGGCAAT	224	55.00	KU922943.1	(Luo et al., 2021) [26]
			54.97		
*SOD*	(F) AGTTTCAGCCGTCTGTTCG (R) CACAGTGCTTACATCACCCTTA	231	58.10	HQ852225.1	(Wang et al., 2021) [29]
			58.06		
*CAT*	(F) GAACTGGGATTTGGTTGGCA (R) GGTCCGAGAAAAGGATGGTG	185	58.66	KC485002.1	(Wang et al., 2021) [29]
			58.26		
*GPx*	(F) CCTGGCTTTCCCCTGTAACC (R) ACCGAGTCATCCGAAGGCA	204	60.32	HQ651155.1	(Wang et al., 2021) [29]
			60.98		
*HSP60*	(F) GTTGCCTTGCTTCGTTGTATGCC (R) GGTAGCAATGGTGTAACACGGCG	120	63.28	KF028596.1	(Wang et al., 2021) [29]
			64.36		
*EIF*	(F) CATGGATGTACCTGTGGTGAAAC (R) CTGTCAGCAGAAGGTCCTCATTA	179	59.56	MH540106.1	(Hu et al., 2018) [30]
			59.80		

Note: *ACC*: acetyl CoA carboxylase; *FAS*: fatty acid synthase; *CPT1*: carnitine palmitoyl-transferase 1; *G6PDH*: glucose-6-phosphate dehydrogenase; *ACBP*: acyl-CoA binding protein; *FABP10*: fatty acid-binding protein 10; *SCD*: stearoyl-CoA desaturase; *SOD*: superoxide dismutase; *CAT*: catalase; *GPx*: glutathione peroxidase; *HSP60*: heat shock proteins 60; *EIF*: eukaryotic translation initiation factor.

**Table 3:** Growth performance of *M. nipponense* after feeding experimental diets for 10 weeks.

Parameters	CT	OF	OF+FA160	OF+FA320
IW (g)	0.140± 0.008	0.140 ± 0.008	0.130± 0.008	0.130 ± 0.007
FW (g)	1.090 ± 0.033 ^a^	0.910 ± 0.025 ^b^	0.990 ± 0.024 ^b^	0.930 ± 0.022 ^b^
SR (%)	79.800 ± 3.693	83.600 ± 1.860	81.400 ± 2.619	78.330 ± 0.989
WGR (%)	707.390 ± 37.102 ^b^	561.510 ± 23.086 ^a^	648.790 ± 35.600 ^ab^	605.140 ± 29.221 ^a^
SGR (%/d)	3.720 ± 0.084 ^b^	3.370 ± 0.063 ^a^	3.590 ± 0.083 ^ab^	3.480 ± 0.072 ^a^
FCR	1.610 ± 0.110	1.840 ± 0.107	1.690 ± 0.095	1.780 ± 0.076
RFI (%/d)	6.440 ± 0.374	6.660 ± 0.205	6.630 ± 0.200	7.080± 0.219

Note: IW: initial weight; FW: final weight; SR: survival rate; WGR: weight gain rate; SGR: specific growth rate; FCR: feed conversion ratio; RFI: relative feed intake; CT: control group; OF: 3% oxidized fish oil; FA: ferulic acid. Data are mean values of six replicates (6 tanks) expressed as mean ± SE. ^a, b^ Within a row, means that are significantly different by Duncan’s test are indicated by different letters (*p <* 0.05).

**Table 4:** Effect of dietary oxidized fish oil with ferulic acid supplementation on hemolymph and hepatopancreas biochemical parameters of *M. nipponense*.

Items	CT	OF	OF+FA160	OF+FA320
Hemolymph				
TG (mmol/L)	0.980 ± 0.056 ^ab^	1.040 ± 0.060 ^b^	0.830 ± 0.038 ^a^	0.850 ± 0.005 ^ab^
TC (mmol/L)	0.660 ± 0.014 ^ab^	0.700 ± 0.031 ^b^	0.550 ± 0.046 ^a^	0.630 ± 0.043 ^ab^
HDL-C (mmol/L)	0.200 ± 0.019 ^bc^	0.150 ± 0.013 ^a^	0.200 ± 0.018 ^c^	0.150 ± 0.009 ^ab^
LDL-C (mmol/L)	0.220 ± 0.007 ^ab^	0.250 ± 0.018 ^a^	0.190 ± 0.013 ^b^	0.200 ± 0.018 ^ab^
TBA (µmol/L)	5.810 ± 0.140 ^b^	5.130 ± 0.273 ^a^	5.960 ± 0.182 ^b^	5.670 ± 0.214 ^ab^
Hepatopancreas				
TG (mmol/gprot)	1.520 ± 0.062 ^ab^	1.830 ± 0.081 ^a^	1.360 ± 0.087 ^b^	1.410 ± 0.251 ^ab^
TC (mmol/gprot)	0.100 ± 0.015 ^a^	0.180 ± 0.041 ^b^	0.110 ± 0.016 ^a^	0.110 ± 0.023 ^a^
HDL-C (mmol/gprot)	0.200 ± 0.032 ^ab^	0.170 ± 0.008 ^a^	0.240 ± 0.014 ^b^	0.210 ± 0.014 ^ab^
LDL-C (mmol/gprot)	0.330 ± 0.026 ^a^	0.460 ± 0.028 ^b^	0.370 ± 0.030 ^a^	0.360 ± 0.035 ^a^
TBA (umol/L/gprot)	5.080 ± 0.253 ^b^	4.210 ± 0.294 ^a^	5.160 ± 0.265 ^b^	5.040 ± 0.188 ^b^

Note: TG: triglyceride; TC: total cholesterol; LDL-C: low-density lipoprotein cholesterol; HDL-C: high-density lipoprotein cholesterol; TBA: total bile acid; CT: control group; OF: 3% oxidized fish oil; FA: ferulic acid. Data are mean values of twelve replicates (2 per tank, 6 replicates) and expressed as mean ± SE. ^a–c^ Within a row, means that are significantly different by Duncan’s test are indicated by different letters (*p* < 0.05).

## Data Availability

The authors confirm that the data supporting the findings of this study are included within the article.
